# Supplementary material for: Eye Movement Desensitization and Reprocessing (EMDR) as a Possible Evidence-Based Rehabilitation Treatment Option for a Patient with ADHD and History of Adverse Childhood Experiences: A Case Report Study
Source: J Pers Med. 2023 Jan 23;13(2):200. doi: 10.3390/jpm13020200 (PMC9961224; doi:10.3390/jpm13020200)
Supplement: Supplementary file 1 [file jpm-13-00200-s001.zip › jpm-2033137-supplementary.pdf]

EMDR therapy is very common around the world. In the United States, the Department of Veterans Affairs and Department of Defense list EMDR as a “best practice” in treating veterans experiencing PTSD. Research on EMDR includes dozens of clinical trials, research studies and academic papers. It has official approval from the World Health Organization (WHO) and government organizations and agencies in the United Kingdom, Australia and Germany, among others.

Only licensed therapists can practice EMDR therapy after certified training by EMDR Institutions, and in our case by EMDR Italia. Dr. Patrizia Brogna is a Level 2 EMDR praticioner eith extensive esperience in treatment of patients with Post-Traumatic Stress Disorders (PTSD).

We used the standard Protocol of EMDR therapy on past memories. EMDR therapy consists of eight phases. These phases occur over multiple sessions, with one session sometimes using parts of several phases. An example of this would be how phases 1 and 2 typically happen only in early sessions, while phases 3 through 8 are part of multiple sessions later. For a single disturbing event or memory, it usually takes between three and six sessions. More complex or longer-term traumas may take eight to 12 sessions (or sometimes more). Sessions usually last between an hour and 90 minutes. The eight phases are reported in Table S1

**Table S1: The eight phases of EMDR therapy**

| | |
|---|---|
| 1 | Patient history and information gathering. This part of the process involves your healthcare provider gathering information about you and your past. This helps them determine if EMDR is likely to help you. It also includes asking about upsetting or disturbing events and memories that you want your therapy to focus on, as well as your goals for this therapy. |
| 2 | Preparation and education. During this phase, your healthcare provider will talk to you about what will happen during EMDR sessions and what you can expect. They’ll also talk to you about things to focus on to help you feel more stable and safer during sessions. They’ll provide you with tools to help you manage your emotions. |
| 3 | Assessment. This part of the process is where your healthcare provider helps you identify themes and specific memories that you may want to work on during reprocessing. They’ll help you identify both negative beliefs about how the trauma has made you feel, as well as positive beliefs that you would like to believe about yourself going forward. |
| 4 | Desensitization and reprocessing. During this phase, your healthcare provider activates your memory by helping you identify one or more specific negative images, thoughts, feelings and body sensations. Throughout the reprocessing, they’ll help you notice how you feel and any new thoughts or insight you have about what you’re experiencing. |
| 5 | Installation. During this phase, your healthcare provider will have you focus on the positive belief you want to build in as you process a memory. This positive belief can be what you said in phase 3 or something new you think of during phase 4. |
| 6 | Body scan. Your healthcare provider will have you focus on how you feel in your body, especially any of the symptoms you feel when you think about or experience the negative memory. This phase helps identify your progress through EMDR therapy overall. As you go through sessions, your symptoms should decrease until you don’t have any (or as close to none as possible). Once your symptoms are gone, your reprocessing is complete. |
| 7 | Closure and stabilization. This phase forms a bridge between later sessions. During this phase, your healthcare provider will talk to you about what you should expect between sessions. They’ll also talk to you about how to stabilize yourself, especially if you have negative thoughts or feelings during the time between sessions. They won’t end a session until you feel calmer and safe. They might also ask you to write down any new thoughts you have about the disturbing event(s), so you can bring them up at your next session. |
| 8 | Reevaluation and continuing care. The final phase of EMDR therapy involves your healthcare provider going over your progress and how you’re doing now. This can help determine if you need additional sessions or how to adjust your goals and expectations for your therapy. They’ll also help you explore what you might experience in the future — how you would like to handle things at that time, knowing what you know now, about yourself and your past trauma. |
